# Modelling the Delta1/Notch1 Pathway: In Search of the Mediator(s) of Neural Stem Cell Differentiation

**DOI:** 10.1371/journal.pone.0014668

**Published:** 2011-02-08

**Authors:** Alexandros Kiparissides, Michalis Koutinas, Toby Moss, John Newman, Efstratios N. Pistikopoulos, Athanasios Mantalaris

**Affiliations:** Biological Systems Engineering Laboratory, Department of Chemical Engineering, Centre for Process Systems Engineering, Imperial College, London, United Kingdom; University of Nottingham, United Kingdom

## Abstract

The Notch1 signalling pathway has been shown to control neural stem cell fate through lateral inhibition of mash1, a key promoter of neuronal differentiation. Interaction between the Delta1 ligand of a differentiating cell and the Notch1 protein of a neighbouring cell results in cleavage of the trans-membrane protein, releasing the intracellular domain (NICD) leading to the up regulation of hes1. Hes1 homodimerisation leads to down regulation of mash1. Most mathematical models currently represent this pathway up to the formation of the HES1 dimer. Herein, we present a detailed model ranging from the cleavage of the NICD and how this signal propagates through the Delta1/Notch1 pathway to repress the expression of the proneural genes. Consistent with the current literature, we assume that cells at the self renewal state are represented by a stable limit cycle and through in silico experimentation we conclude that a drastic change in the main pathway is required in order for the transition from self-renewal to differentiation to take place. Specifically, a model analysis based approach is utilised in order to generate hypotheses regarding potential mediators of this change. Through this process of model based hypotheses generation and testing, the degradation rates of Hes1 and Mash1 mRNA and the dissociation constant of Mash1-E47 heterodimers are identified as the most potent mediators of the transition towards neural differentiation.

## Introduction

**Figure 1:**
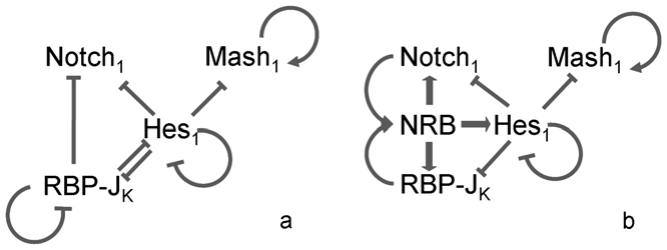
The gene cascade controlling cell fate in neural progenitor cells as modelled herein (a) in the absence and (b) in the presence of a *delp* signal. (>) denotes up-regulation (|) denotes repression. The proneural genes (*mash1*) activate the transcription of genes that promote differentiation towards neural cells. On the other hand, some bHLH genes (*hes1*, *notch1*, *rbpj*) inhibit commitment of neural progenitor cells either by intersecting the formation of E box binding heterodimers and/or by forming homodimers that bind with high affinity to specific DNA sequences termed N boxes. The presence of a delta signal from a differentiating neighbouring cell causes the cleavage of the intracellular domain of membrane bound Notch protein. The NICD travels into the nucleus where it forms a complex with RBPJ (denoted as NRB). This complex binds on the RBPJ sites of *hes1*, *notch1* and *rbpj* and induces transcription.

The correct timing and distribution of differentiation in neural stem cells is critical for the integrity, shape, and size of the developing brain and the proper functioning of the central nervous system in mammals [1]–[5]. In addition to its significance in the development of the CNS and PNS, Notch signalling is responsible for the development of several tissues and organs during embryo development [1], [2], [6], [7], [8]. Furthermore degenerative brain diseases, such as Alzheimer's disease, have been linked [9] to malfunctions of the Notch signalling pathway. The process of commitment to the neurogenic lineage is directed by a cascade of antagonistic basic – helix – loop – helix (bHLH) genes evolving around the Delta1/Notch1 signalling pathway. The activator type genes (*mash1*, *hes6*) usually form heterodimers with ubiquitously expressed proteins, such as E47, which bind specific DNA sequences (CANNTG) termed E boxes. E box binding activates the transcription of genes that promote differentiation towards neural cells. The repressor type bHLH genes, which include members of the Hairy/Enhancer of split family (*hes1, 3, 5*), inhibit commitment of neural progenitor cells either by intersecting the formation of E box binding heterodimers and/or by forming homodimers that bind with high affinity to specific DNA sequences (CACNAG) termed N boxes. N box binding represses the transcription of the bearing gene. Though a number of genes have been associated with the Delta1/Notch1 signalling pathway, the most dominant ones are the repressor genes *hes1*, *notch1*, *rbpj* and the activator gene *mash1* [2], [4], [6], [10] shown in figure (1).

Both the activator and repressor type genes are active from the very early stages of development [11]. However, *hes1* activity has been shown to decrease the more a cell commits towards a specific cell type while Mash1 is up-regulated [4], [11], [12], [13]. During the neural progenitor phase Hes1 expression is higher than Mash1 expression and it has been shown that the concentrations of these proteins oscillate with a constant period of roughly 2 h [14]. Commitment to specific lineages alleviates the oscillatory behaviour typical of the Delta1/Notch1 pathway and results in down-regulation of Hes1 expression while Mash1 is up-regulated [4]. These observations indicate that Hes1 is a crucial element in the cell fate decision process; however the molecular mechanisms that govern the transition from progenitor cells to specific neural lineages remain unknown [4].

The oscillatory behaviour of the Notch signalling pathway has been the focus of a number of modelling studies following publication of experimental findings [14]. A number of approaches has been attempted, starting with the use of time-delayed differential equation systems [15], [16], [17], [18], feedback differential equation models [14], [19], and more recently stochastic models [20], [21]. Most of the modelling attempts focus on the Hes1 oscillator and disregard the remainder of the signalling pathway in an attempt to remain tractable. Nevertheless, they manage to capture the experimental observations satisfactorily and derive a number of conclusions from the numerical simulations performed.

Hirata et al. [14] report that a simple negative feedback loop in which *hes1* transcription is repressed via its own protein product is not adequate to reproduce sustained oscillations. They successfully reproduce the experimental results via the inclusion of an “unknown” factor which they speculate is either a chemical or environmental parameter. Jensen et al. [15] conclude that the oscillatory behaviour is mainly controlled through the degradation rates Hes1 mRNA and Hes1 protein via the activity of proteases. Monk, [17] further supports this notion while also indicating the need to further elucidate the effectors for the transcriptional delay in negative feedback oscillatory systems. Later work by Veflingstad et al. [22] further supports that mechanisms other than the regulation applied by the Delta1/Notch1 pathway at the transcriptional level may mediate its oscillatory behaviour and interestingly highlights protein interactions as possible effectors. Momiji and Monk [18] perform an exhaustive dynamic analysis on the effect of local feedback loops in a model of lateral inhibition based on the Notch signalling pathway and are the first to explore the prerequisites for neuronal differentiation. By manipulating the delays in their time delayed model, they are able to show that dampened oscillations and up-regulation of proneural proteins in one cell lead to down-regulation of the same proteins in a neighbouring cell.

Agrawal et al. [20] present the most up to date and complete mathematical description of the Notch signalling pathway, initiating from the cleavage of the intracellular domain of the Notch1 protein (NICD) all the way to expression of Hes1 protein regulated via RBPJ and itself. Their model includes both a deterministic and a stochastic description of the pathway and proves mathematically that the Notch signalling pathway can act both as a bistable switch and as an oscillator depending on the level of repression Hes1 homodimers apply on the N box sites (via a parameter termed rbox). This further hints to the existence of a mechanism by which the repressive effects of Hes1 are alleviated via an extrinsic, to the pathway, signal. However, due to the exhaustive description of all the possible binding site occupancies the model is rather complex and moreover lacks a description of the neural differentiation promoting genes (*mash1*, *hes6, dll1*).

Herein, we present the formulation of a detailed mathematical model that is used to qualitatively investigate the dynamic characteristics of the Delta1/Notch1 pathway which ultimately determines the fate of neural stem cells. Based on the assumption that cells at the self renewal state are represented by a stable limit cycle, we present a detailed, up-to-date mathematical description of the Delta1/Notch1 signalling pathway and demonstrate the behaviour of the system not only during the oscillatory phase but also during the transition towards specific cell lineages and at the differentiated state. It is plausible that the signal that instigates the behavioural change leading to the down-regulation of Hes1 is either the result of cross-talk with a different signalling pathway [3], [6], [7], [23] or the result of the combined effect of many processes [24]. Herein we will test this hypothesis through *in silico* experimentation and model based hypothesis generation. The need for a signal that instigates the behavioural change that leads to neural differentiation is demonstrated and potential mediators of this signal are explored through model analysis techniques.

## Methods

Zeiser et al. [19] presented an adaptation of the Goodwin [25] model for the Hes1 oscillator showing that it was a valid platform for its description. However, their model only considered the behaviour of Hes1 and its mRNA which led to high values for the Hill coefficients. The model developed herein incorporates all major components of the Notch signalling pathway, as described below, in an effort to study the behaviour of the delta/notch pathway in a single cell in the presence or absence of a delta signal from a neighbouring cell.

### Model Formulation

All genes were modelled based on a modified version of the model originally presented by Goodwin [25]. The cell is divided in two compartments facilitating the monitoring of nuclear and cytoplasmic concentrations. In the case of mRNA this compartmentalisation allows the model to account for the delay between transcription and initiation of translation due to a number of processes involved in between including, splicing and translocation. In the case of proteins the compartmentalisation enables the model to account for the different concentrations inside and outside of the nucleus while also taking into account time delays between protein synthesis and post-translational modification. The general formulation followed herein for the description of each gene is presented in equation (1) below:
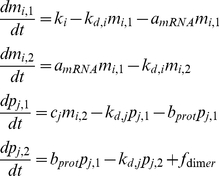
(1)


Where m_i,x_ and p_j,z_ denote the concentrations of the *x*-th mRNA instance of gene *i* and the *z*-th instance of protein *j*, respectively. C_j_ denotes the specific translation rate of protein *j* from its respective mRNA transcripts of gene *i*. k_d,i_ and k_d,j_ denote the degradation rates of the mRNA molecules of gene *i* and protein *j*, respectively, while a_mRNA_ and b_prot_ are the respective transfer constants between the various compartments. The term *f_dimer_* was added in order to account for the depletion of the proteins that form heterodimers.

k_i_ denotes the transcription rate of the *i^th^* gene and is formulated as a maximum theoretical transcription level k_i,0_ modified by the nuclear concentrations of its activators and its repressors as shown below by equation (2):
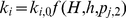
(2)


Function *f_i_(H,h)* as introduced by [25] and later applied by [19] is a Hill function where H denotes the Hill constant and h denotes the Hill coefficient, respectively. In order to account for inhibition by multiple proteins on the transcription of mRNA of gene *i*, we have generalised function *f_i_*, as shown by equation (3):
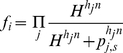
(3)


Parameter *n* was introduced in order to differentiate between repressors binding only as dimers and repressors that have the ability to bind as single molecules. When repressor *j* binds on gene *i* as a dimer, then parameter *n* is equal to 2; if the repressor binds as a single molecule, parameter *n* is equal to 1. The Hill coefficient *h*, takes its value according to the number of binding sites for repressor *j* present in gene *i*. The resulting exponents are high and similar to the ones observed by other studies [19], however based on the rationale presented above we have assigned a mechanism that justifies such values. It is worthwhile mentioning that lower exponents can not sustain oscillatory behaviour indicating highly non-linear and complex interactions that take place while the cells are at the undifferentiated state. Another possible explanation is that the exclusion from the model of parts of the Delta1/Notch1 pathway that are not fully understood renders the use of higher exponents a necessary means to compensate for the lack of mechanistic information.

Hes1 has been shown to oscillate with a 2 h period and is known to inhibit its own transcription [14]. Hes1 protein forms homodimers which bind specific DNA regions, the N boxes. According to [20] there are 3 N box binding sites contained within the 2,463bp long gene which can repress Hes1 mRNA transcription up to 40 times. Furthermore, *hes1* has been shown to have 2 adjacent RBPJ binding sites. RBPJ is known to function both as a repressor and as an activator when NICD is present [4], [20]. Another member of the HES gene family identified recently, *hes6*, has been shown [10], [11], [23] to alleviate the repressive effects of Hes1 on *mash1* transcription. Hes6 can form heterodimers with Hes1, thus sequestering Hes1 molecules and prohibiting the formation of Hes1 homodimers that repress *mash1* transcription. Based on these observations equations (4) were formulated in order to describe the dynamics of the transcription of Hes1 mRNA, the formation of Hes1 proteins and the formation of Hes1-Hes6 heterodimers.
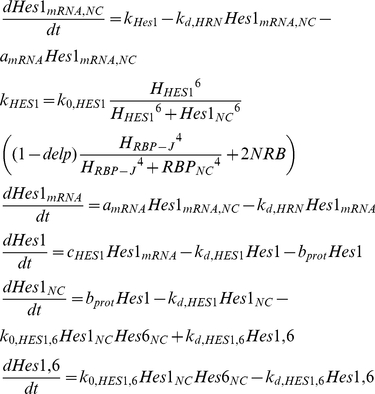
(4)


d*elp* is a binary variable used to denote the existence or absence of a delta signal from a neighbouring cell. NRB denotes the concentration of the NICD-RBP complex in the presence of a delta signal and the equations describing its formation are presented along with the equations describing the cleavage of the NICD further below. The multiplier preceding the concentration of the NRB complex equals the number of RBP binding sites on the respective gene.

*Rbpj* gene has been reported to be ubiquitously expressed [26] or regulated by the protein Hairless [27] in *Drosophila*. However, based on sequence motif recognition software, [20] modelled the Delta1/Notch1 pathway under the assumption that *rbpj* itself is repressed by the Hes1 homodimers containing 3 N box binding sites and 3 RBPJ binding sites. Herein we will follow the assumption made by [20] under the rationale that even if RBPJ is ubiquitously expressed, compartmentalisation, competition with other proteins and concentration gradients might effectively limit the amount of available RBPJ for interaction with the elements of the studied pathway. Similarly to the *hes1* gene, we have modelled the *rbpj* transcription to be inhibited by RBPJ in the absence of NICD, yet to be promoted by the NICD-RBPJ complex. As NICD concentration increases, so does the amount of NICD-RBPJ complexes being formed resulting in a smaller pool of “free” RBPJ proteins.
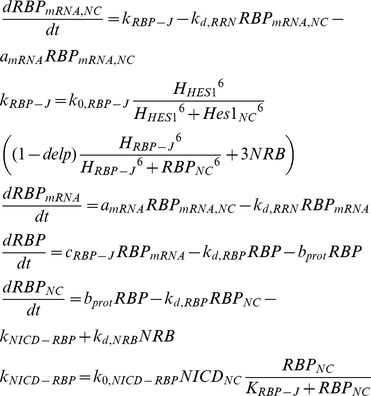
(5)


Similarly, the *notch1* gene has been shown to have one putative N box site and two putative RBPJ sites [20].
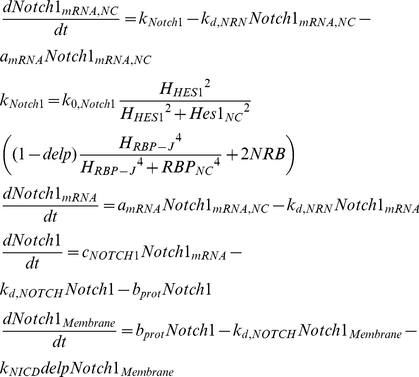
(6)


These 3 genes (*notch1*, *hes1*, *rbpj*) form a complex loop of negative regulation applied on each other (figure 1). While neural stem cells remain in the neural progenitor state, the net effect of this gene cascade is to repress the expression of the neuronal differentiation inducing genes (*mash1*, *hes6*). Mash1 is a bHLH protein that has been shown to induce the expression of genes that promote neuronal differentiation [4], [11], [13]. Mash1 forms heterodimers with the ubiquitously expressed *E* proteins, like E-47, which bind on the E-boxes of the neuronal differentiation inducing genes and enhance transcription of these genes [12]. The 2,828bp sequence of *mash1* contains one N box site [28] which results in its inhibition by Hes1 homodimers. Furthermore, Hes1 has been shown to have a negative effect on the ability of Mash1 to form heterodimers with E-47 [12]. Upon dissociation of the heterodimer, Mash1 is not re-added to the pool of available Mash1 since it has been shown [29] that during the heterodimerisation process with E-47, Mash1 undergoes phosphorylation which effectively alters its structure.
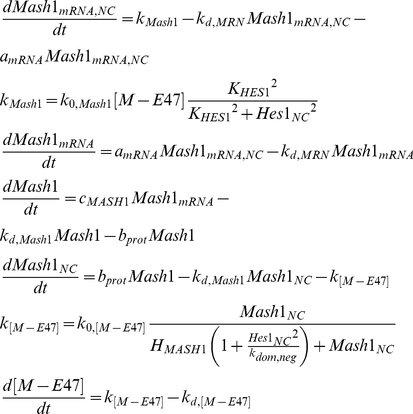
(7)


By sequestering Hes1 proteins, Hes6 enhances the formation of Mash1/E47 dimers [13] which have been demonstrated to bind on the E box site contained in the *mash1* gene and induce transcription. Up-regulation of *mash1* transcription results in a consequent up-regulation of the downstream neural differentiation promoting genes.
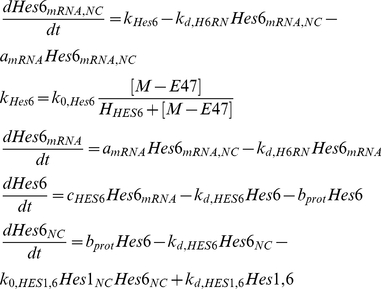
(8)


The presence of a delta signal from a differentiating neighbouring cell causes the cleavage of the intracellular domain of membrane bound Notch protein. The NICD travels into the nucleus where it forms a complex with RBPJ (denoted as NRB). This complex binds on the RBPJ sites of *hes1*, *notch1* and *rbpj* and induces transcription. Herein we have assumed that the transfer of the NICD to the nucleus follows saturation kinetics when NICD is present in abundance. This simplification was made in order to compensate for the absence of information regarding the regulation of the cleavage of NICD. Upon dissociation from the NRB complex, NICD is targeted for proteolysis, therefore it is not added to the free NICD pool. Herein we have modelled the presence of a delta signal from a differentiating neighbouring cell as a binary variable that is 1 if a delta signal is present and 0 if otherwise.
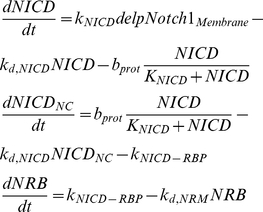
(9)


### Model Parameters

Due to the limited availability of dynamic experimental measurements of the modelled variables, the nature of the model is qualitative rather than quantitative. Nevertheless the choice of parameter values is not trivial as it ultimately defines the behaviour of the model. [30] encountered the same lack of detailed information when studying the circadian clock in *Arabidopsis thaliana*. In their work they conclude that any form of quantitative comparison between their genetic network model and any set of experimental mRNA traces would be inappropriate due to the sparsity and inherently noisy nature of such data. Instead they formulate an empirical cost function which they evaluate over a large number of random points, accounting for varying parameter values, and declare the set that minimises the value of the cost function as the optimal parameter set. The formulation of the cost function was a sum of terms that quantify the agreement between the model output and an experimentally observed qualitative feature. Following this approach consisted of running the model initially with parameter values taken from relevant literature where available, or estimates of the appropriate order of magnitude where not available; in order to verify that it satisfactorily captures the behaviour of the modelled system.

**Figure 2:**
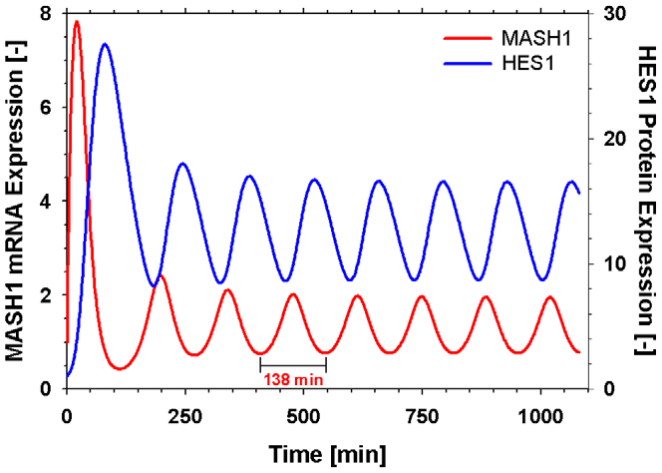
Oscillatory expression of Mash1 at steady state for non-differentiated cells using the parameter values from Table 1. Oscillatory behaviour of the pathway with a period of roughly 2.5 h. Mash1 concentration peaks between the concentration peaks of its repressor Hes1.

According to [17], delayed feedback drives oscillations only if the relevant mRNA and protein half-lives are sufficiently small relative to the delay. We have, therefore, considered the same degradation rates for the mRNA and proteins of all genes equal to those estimated experimentally for the Hes1 protein [14] and utilised in a number of studies [15], [17], [20], [21]. From a biological point of view this might be an oversimplification; however, from a mathematical point of view it strongly supports oscillatory behaviour. Quantitatively this simplification only affects the period of oscillation. The parameters for the Hill functions and the mRNA and protein transfer constants were taken from [20] where a similar model structure is followed. The model was simulated with the parameter values presented in Table 1 and was able to describe the oscillatory behaviour of the pathway with a frequency of roughly 2.4 h, which is slightly higher that the reported approximately 2 h period in fibroblasts [14]. This can be attributed to the assumption that the mRNA and proteins of all genes were appropriated the same degradation rates. Figure 2 shows the oscillatory behaviour of Mash1, whose concentration peaks, as would be expected, between the concentration peaks of its repressor, Hes1.

**Table 1:** Initial Parameter values.

Parameter	Value	Description	Source
k_d,HRN_, k_d,RRN_, k_d,NRN_, k_d,MRN_, k_d,H6RN_	0,028	mRNA degradation rate (min^−1^)	[14]
k_d,HES1_, k_d,RBP_, k_d,NOTCH_, k_d,MASH_, k_d,HES6_	0,031	protein degradation rate (min^−1^)	[14]
a_mRNA_	0,05	mRNA intracellular transfer rate (min^−1^)	[19]
b_prot_	0,05	protein intracellular transfer rate (min^−1^)	N/A
c_i_	0.2	mRNA translation rate for protein *i* (min^−1^)	[19]
H_i_	10	Hill coefficient for gene *i* (M^−1^)	[19]
k_dom,neg_	15	Constant for Hes1dominant negative repression on Mash1 transcription (M)	N/A
k_0,Hes1_, k_0,Mash1_, k_0,RBP-J_, k_0,Notch1_, k_0,Hes6_	1	Transcription constant for Hes1 and Mash1(min^−1^)	N/A
k_0,[M-E47]_, k_0,Nicd-RBP_	0.1	Constants for MASH1-E47 & Nicd-RBPJ complex formation (min^−1^)	N/A
k_0,HES1,6_	0.001	Constant for Hes1-Hes6 complex formation (min^−1^)	N/A
k_d,NRB_	0.003	Constant for Nicd-RBPJ complex dissociation (min^−1^)	N/A
k_d,[M-E47]_, k_d,HES1,6_	0.031	Constants for MASH1-E47 & Hes1-Hes6 complex dissociation (min^−1^)	[14]
k_d,NICD_	0.0385	protein degradation rate (min^−1^)	N/A
K_RBP-J_, K_NICD_	1	Hill coefficient (M^−1^)	N/A

Following the approach of [30] in order to derive the optimal parameter values the model was evaluated over a total of 2^14^ randomly generated points each representing a vector of all parameter values. The random points were generated using the Sobol [31] quasi-random number generator which is regarded by many [32], [33], [34], [35] the best in uniformly filling hypercubes of large dimensionalities. The parameters involved in the generation and translocation of the NICD (*k_NICD_, K_NICD_, K_RBP-J_, k_d,NICD_, k_0,NICD-RBP_, k_d,NRB_*) were excluded from this process as they require the presence of an active delta signal which results in a deviation from the oscillatory resting state for which experimental data are available. Furthermore, appropriating the same transfer constants (*a_mRNA_, b_prot_*) for all modelled variables is a simplification of the model which was done in an attempt to reduce the size of the parameter vector and hence the dimensionality of the parameter space that needs to be sampled. The values used herein have been taken from the work of [19] on a similar model, albeit studying merely the Hes1 gene by itself. Thus these two parameters have also been excluded from the optimal parameter search.

**Figure 3:**
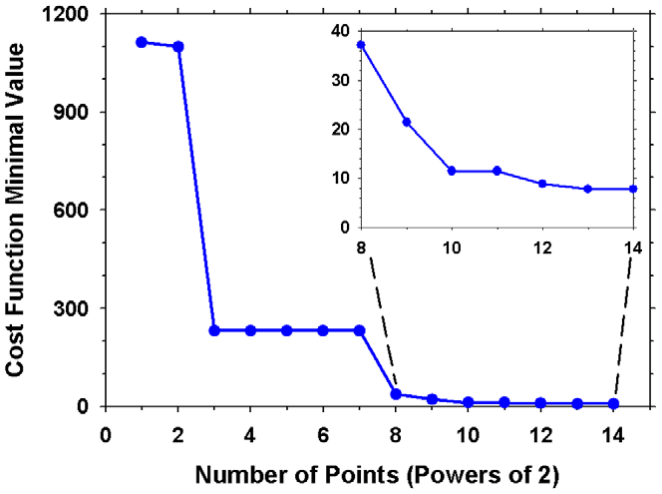
Convergence of the minimum value of the empirical cost function with the number of Sobol points.

This resulted in a vector of 31 parameters which were randomly varied within ±90% of their initial value in search of the optimal parameter set. The model was simulated for 3,000min allowing enough time for a resting state to be reached and the cost function was evaluated over the last 1,000min in order to ensure that the resting state has been reached. Details on the formulation of the cost function can be found in Supplemental Material (S1). Figure 3 displays the evolution of the minimal value of the cost function as the number of sampled points increase. The minimum of the cost function slowly converges towards a final value after 2^12^ points. The computational cost of increasing the number of sampled points beyond 2^14^ is prohibitive and counterintuitive when taking into consideration the limited benefits of doing so. Thus the optimal parameter set as identified by this search of the parameter space can be found in Table 2.

**Table 2:** Optimal parameter values.

Parameter	Optimal Value	Parameter	Optimal Value
k_d,HRN_	0.0363	k_0,NOTCH1_	1.5926
k_d,RRN_	0.0242	k_0,MASH1_	0.3876
k_d,NRN_	0.0390	k_0,[M-E47]_	0.0630
k_d,MRN_	0.0485	k_0,HES6_	1.0749
k_d,H6RN_	0.0520	k_0,HES1,6_	0.0002
k_d,HES1_	0.0379	c_HES1_	0.3635
k_d,RBP_	0.0233	c_RBP-J_	0.0969
k_d,NOTCH_	0.0311	c_NOTCH1_	0.0820
k_d,MASH1_	0.0070	c_MASH1_	0.1049
k_d,HES6_	0.0079	c_HES6_	0.3594
k_d,[M-E47]_	0.0581	H_HES1_	3.7486
k_d,HES1,6_	0.0289	H_RBP-J_	1.9378
k_d,NRB_	0.0028	K_HES1_	7.9462
k_dom,neg_	18.6882	H_MASH1_	5.1724
k_0,HES1_	1.0056	H_HES6_	17.4502
k_0,RBP-J_	0.3867		

## Results

**Figure 4:**
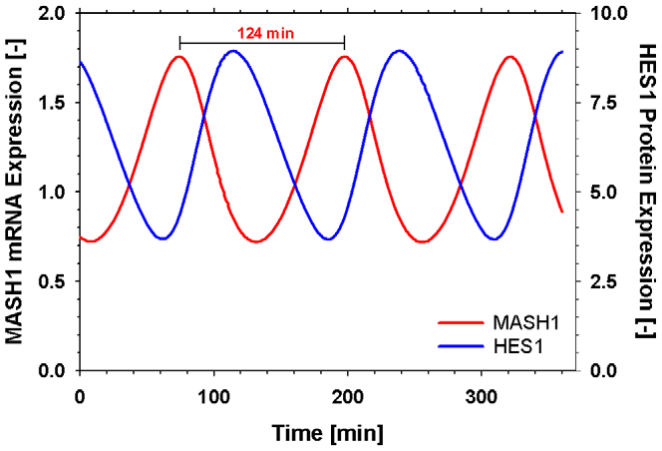
Oscillatory expression of Mash1 at steady state for non-differentiated cells using the optimal parameter values from Table 2. Oscillatory behaviour of the pathway with a period of 2.0h. Mash1 concentration peaks between the concentration peaks of its repressor Hes1.

The model was simulated with the parameter values presented in table 2 and was able to describe the oscillatory behaviour of the pathway with a frequency of roughly 2h, in accordance to experimental observations [14]. Figure 4 shows the oscillatory behaviour of Mash1 with the concentration peaks occurring between the concentration peaks of its repressor Hes1.

### Application of a Delta signal from a neighbouring cell

**Figure 5:**
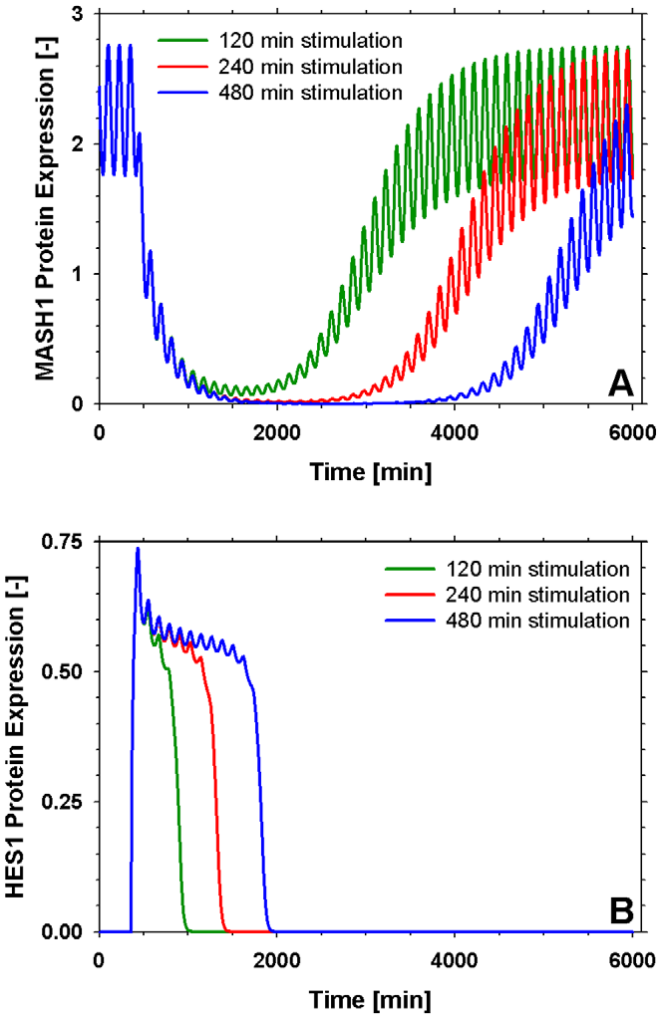
Response to the application of a delta signal for varying time periods. (A) Mash1 and (B) Nicd expression. As the duration of the application of the delta signal increases so does the lag period required for the system to return to its original resting state. The duration of the application of the delta signal has no effect on the maximal NICD concentration.

As mentioned previously, under the effect of a Delta signal from a neighbouring cell, the upregulation of the main effecter of the Delta/Notch pathway, *hes1*, results in the overall repression of the neuronal differentiation inducing genes (*mash1, hes6*). Therefore we applied a delta signal for varying amounts of time and studied the response of the key components of the pathway. Figure 5 displays the concentration of Mash1 protein as a response to the application of a delta signal for varied amounts of time. In accordance to experimental observations, Mash1 is downregulated in the presence of a delta signal. As the duration of the delta signal increases so does the lag phase upon termination of the signal until Mash1 reaches its original resting state. Figure (S1) showcases the effects of the delta signal on Hes1, Mash1 and Hes6. It is worth mentioning that the level of upregulation of Hes1 seems to reach a plateau after a certain duration of the delta signal which is in agreement with the observations of [20].

### The need for an extrinsic signal

**Figure 6:**
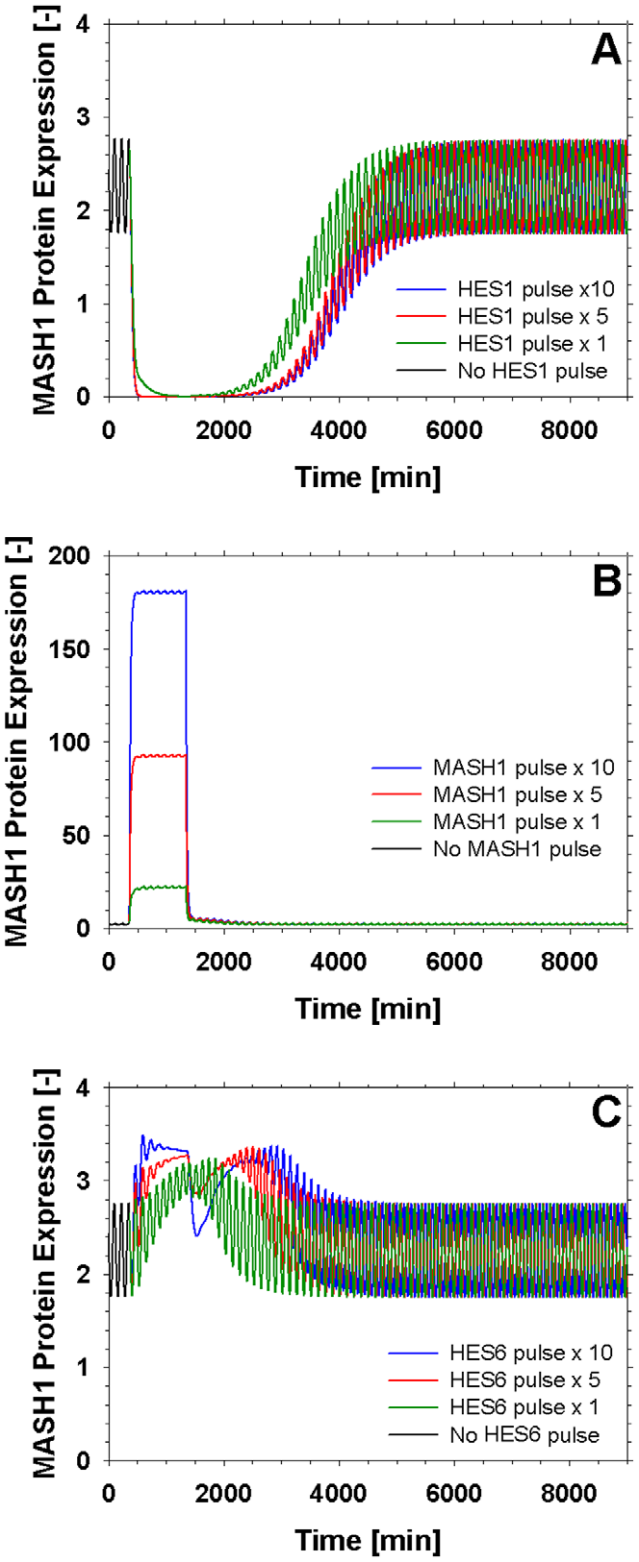
Response of Mash1 expression to the application of variably sized pulses (×1, ×5, ×10) for a fixed time period (980min). The pulse was applied through a constant generation term in the right hand side of the equations describing cytoplasmic Hes1 (A), Mash1 (B) and Hes6 (C) concentrations respectively.

Subsequently the robustness of the gene network was tested by applying variably sized pulses for a fixed amount of time (980min) to various components of the pathway and studying the response. The model proved robust to pulses in the concentrations of Hes1, Mash1 and Hes6, as shown in Figures 6 and (S2), (S3) and (S4). Initially, the effect of a variably sized (×1, ×5 and ×10) pulse in the concentration of Hes1 on the system was investigated. The pulse was applied through a constant generation term in the right hand side of the equation describing cytoplasmic Hes1 concentration. The system proved resilient to the application of such a pulse and always returned to the original oscillatory resting state. In order to further study the stability of the system under disturbance phase plane graphs of Hes1, Mash1 and Hes6 mRNA transcripts versus their respective protein products were generated for the pulse experiments (Figures S2, S3, S4), which further prove the model's inertia against a new resting state. Notably only the pulse in Hes6 (and not Mash1) concentration had a significant effect on the levels of Hes1 (Figure S4). This behaviour is in agreement with the experimental findings of [3], [6], [36]. This behaviour confirms [15], [17] that cells in the self-renewal state are resilient against concentration changes of the elements that constitue the Notch signalling pathway supporting part of our hypothesis that the influence of a signal extrinsic to this pathway is required in order to drive cells towards neuronal differentiation.

### A model based hypothesis generation approach

A model based approach was employed in order to identify components of the Delta/Notch pathway as viable candidates able to mediate the required behavioural change which results in neuronal differentiation. In the context of our assumption that cells in the self renewal state are in a stable limit cycle, differentiation would be equivalent to a new non-oscillating resting state. As shown in previous studies [37], [38], [39] a systematic model based approach can provide biological insight and information to the experimentalist. Model analysis techniques, such as Global Sensitivity Analysis (GSA), can provide behavioral information regarding the hierarchical structure of the modeled system that would otherwise be hard to extract from experimental observations alone [40]. GSA allocates the uncertainty in the model output to the various sources of uncertainty, namely the model parameters. Usually a small number of parameters accounts for the majority of the uncertainty observed in the model output whereas the majority of parameters have little to no effect on the model output when varied within a certain range [38]. Herein we employ GSA in order to identify the parameters with a stronger effect on the behaviour of the studied network. Subsequently we study how varying the value of these parameters affects the behaviour of the model.

**Figure 7:**
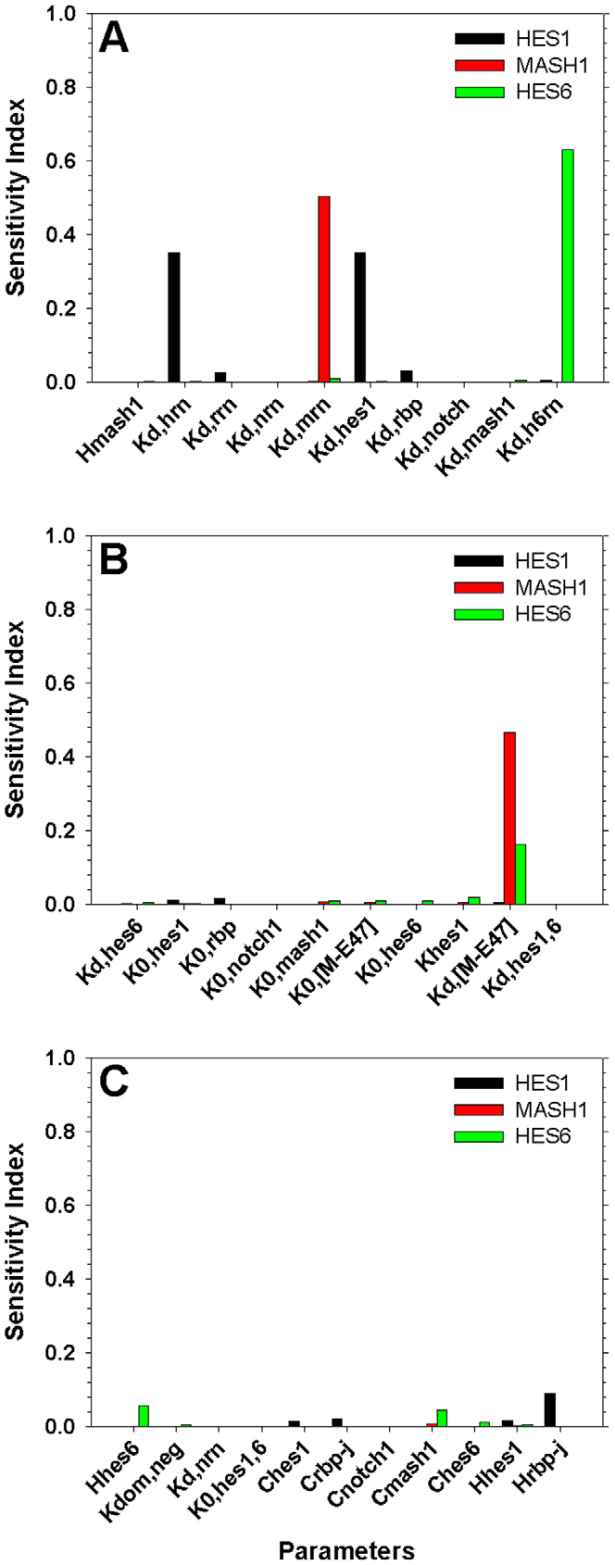
Results of Global Sensitivity Analysis. Model parameters were varied within ±90% of their optimal value and their effect on the model output (black bars – Hes1, red bars – Mash1, green bars – Hes6) was analysed using the Derivative Based Global Sensitivity Measures.

Derivative Based Global Sensitivity Measures [41] (Supplemental Material S1) was chosen as the most appropriate GSA method, as it has been proven ideal for the scanning of medium sized non-linear models [38] due to its computational efficiency. The GSA focused on the 31 model parameters that were optimised using the approach of [30] as described previously. All parameters were varied within ±90% of their optimal value and GSA was performed after 2,000min of model time, allowing for any major disturbances to be alleviated. The outputs studied were the protein concentrations of Hes1, Mash1 and Hes6. Table 3 summarises the results of Figure 7. As expected the majority of the variation observed in the studied outputs can be attributed to 5 out of the total of 31 model parameters. Based on the GSA results we postulate that by varying one or more of these parameters we can achieve a significant shift in the pathway's behaviour resulting in a new resting state typical of differentiated cells. Moreover, parameters with a sensitivity index lower that 0.1 have little to no effect on the model output, thus increasing our confidence on the chosen parameter values. It is interesting to note that all of the significant parameters were either decay or dissociation rates which is in agreement with the conclusions of previous studies [14], [15], [17]. Having identified the most significant parameters with respect to the model output we then performed a series of *in silico* experiments to investigate whether these parameters can mediate a behavioural change towards neural differentiation, when affected one-at-a-time or in pairs of two. The parameters of Table 3 were varied linearly by up to a factor of 10 or until the concentration of Hes1 reached a significantly small value, indicating that Hes1 had indeed been down regulated both in the absence (subscripted with 1 in figures) and in the presence (subscripted with 2) of a delta signal from a neighbouring cell.

**Table 3:** GSA Results – Significant Parameters.

Parameter	Range Examined
k_d,HRN_	0.0363±90%
k_d,MRN_	0.0485±90%
k_d,HES1_	0.0379±90%
k_d,H6RN_	0.0520±90%
k_d,[M-E47]_	0.0581±90%

### Hes1 mRNA degradation (k_d,hrn_)

**Figure 8:**
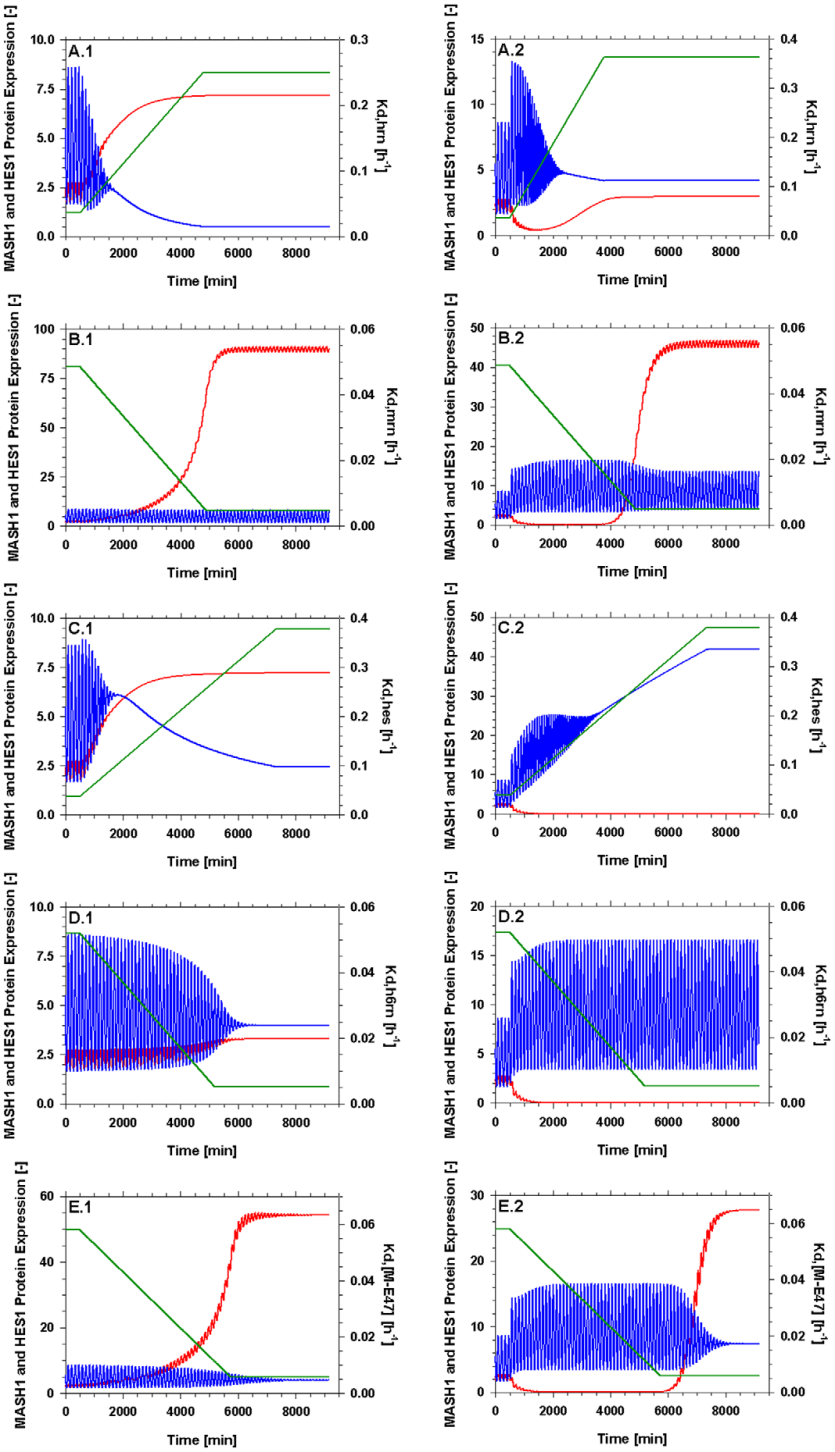
Dynamic evolution of the pathway while varying the value of the parameters of table 3 one-at-a-time linearly by a factor of 10 both in the absence (subscripted with 1) and the presence (subscripted with 2) of a delta signal from a neighbouring cell. The effect of an: (A) increase in the value of k_d,HRN_; (B) decrease in the value of k_d,MRN_; (C) increase in the value of k_d,HES_; (D) decrease in the value of k_d,H6RN_; (E) decrease in the value of k_d,[M-E47]_.

In support of our model-driven hypothesis, [42] identified a type of micro-RNA that specifically binds Hes1 mRNA and induces *hes1* silencing at the post-transcriptional level. They further comment that the presence of this mRNA regulator is crucial for the neuronal differentiation of NT2 cells. Since our model doesn't include such a regulatory mechanism this could be implemented by an increase in the value of Hes1 mRNA degradation accounting for the function of this mi-RNA. Figure 8.A.1 confirms this behaviour as an increase in the value of Hes1 mRNA degradation rate eventually resulted in the down regulation of Hes1 and the up regulation of the neuronal differentiation gene Mash1. On the contrary, in the presence of a delta signal from a neighbouring cell (Figure 8.A.2), this action alone is not adequate to mediate a sufficient down regulation of Hes1. While oscillations are dampened and Mash1 is slightly up regulated, Hes1 is still expressed at a higher level than Mash1. According to [43] however, an up regulation of Notch1 results in the significant down regulation of miR-326 which has been linked with the regulation of the effectors of the delta/Notch pathway. Essentially this translates into the fact that the differentiation suppression signal of dll1 is perhaps stronger than the differentiation promoting activity of mi-RNAs althought, to our knowledge, this hasn't been experimentally validated.

### Mash1 mRNA degradation (k_d,mrn_)

A number of experimental studies have reported that a potential increase in the stability of Mash1 mRNA can be crucial in mediating neuronal differentiation. More specifically, [44] report that the up regulation of the co-activator/repressor Tripp15/CNS2 leads to the differentiation of P19 cells even in the absence of Retinoic Acid (RA) stimulation. They observe no increase in the levels of Mash1 mRNA but an increase in Mash1 protein levels. [45] have recently investigated the role of the histone demethylase Jmjd3 in neuronal differentiation. They conclude that Jmjd3 is crucial for the up regulation of Mash1 in P19 cells under RA stimulation through knock out experiments. [46] show the significance of miR-124 during the neuronal differentiation in P19 cells. One would therefore expect that an increase in the stability of Mash1 mRNA should increase Mash1 protein levels and furthermore promote neuronal differentiation. Implementing this in our model through a reduction in the Mash1 mRNA degradation rates, results in the above described behaviour. Mash1 mRNA levels, are slightly increased (5-fold increase, data not shown) while Mash1 protein levels are increased by a factor of 50 (Figure 8.B.1). This obviously results in a down regulation of Hes1 protein; however the oscillatory behaviour in both proteins is maintained. This could be an indication that this change alone is not enough for a complete down regulation of Hes1 expression which is typical of differentiating cells. This behaviour is expected according to the experimental studies mentioned above, as they all required RA stimulation along with the up regulation of the neuronal differentiating factors in order to achieve differentiation. Under the effects of a delta signal (Figure 8.B.2) this behaviour is even more evident, even though the model doesn't include a degradation term for Mash1 protein in the presence of NICD [47].

### Hes1 protein degradation (k_d,hes_)

Many of the mathematical studies of the oscillatory behaviour of the *hes1* gene and its protein product have already confirmed the significance of the degradation rate of Hes1 protein in the presence or absence of oscillations [14], [15], [17]. This is further confirmed by the experimental work of [14] stating that an increase in the degradation of Hes1 would result in an increase in Hes1 mRNA levels and depletion of Hes1 protein. When implemented in our model, through an increase in the Hes1 protein degradation rate (Figure 8.C.1) this behaviour is successfully reproduced and leads to an up regulation of Mash1 protein in the absence of a delta signal. However, when a delta signal is present (Figure 8.C.2) the effect of the increased degradation of Hes1 is not adequate to alleviate the up regulation of Hes1 protein levels and the resulting down regulation of Mash1.

### Hes6 mRNA degradation (k_d,h6rn_)

Apart from its role in the sequestration of Hes1 monomers, Hes6 has been linked to cell cycle regulation [48]. In fact, over expression of Hes6 mRNA has been reported [49] to disrupt normal differentiation rather than promote it. Implementing an over expression of Hes6 in our model, through a decreased mRNA degradation rate (Figure 8.D.1), results in a down regulation of Hes1 protein, a slight up regulation of Mash1 protein and a concomitant dampening of the oscillations. However on its own, either in the absence or presence of an active delta signal (Figure 8.D.2), the effect of Hes6 over expression is not able to promote neural differentiation. [50] conclude that the altered behaviour in the expression of Mash1 which essentially leads to neural differentiation, could be attributed to the function of Hes6; however this has not been experimentally validated.

### Mash1 – E47 dimmer dissociation (k_d,[M-E47]_)

BMP-2 induces a post-transcriptional decrease in Mash1 levels through enhanced degradation. While studying Mash1 stability under the effects of BMP-2 over expression [29] reported that over expression of E47 significantly up regulates Mash1 protein concentration even under the effects of increased BMP-2 levels. Furthermore [51] reported that co-expression of neural proteins Nng2 and Mash1 with E47 proteins resulted in neural stem cell differentiation and protection of these proteins from the repressive effects of the Delta/Notch pathway. Implementing this in our model through a decrease in the Mash1 – E47 dimmer dissociation constant resulted in a significant up regulation of Mash1 and a down regulation of Hes1 concomitant with the dampening of the oscillations both in the absence (Figure 8.E.1) and in the presence of a delta signal (Figure 8.E.2). It is worthwhile mentioning that in the presence of a delta signal, Hes1 protein levels were not significantly reduced despite the increase in Mash1 protein. This could indicate that the change in the dimer dissociation constant alone is not an effective mediator of neural differentiation.

**Figure 9:**
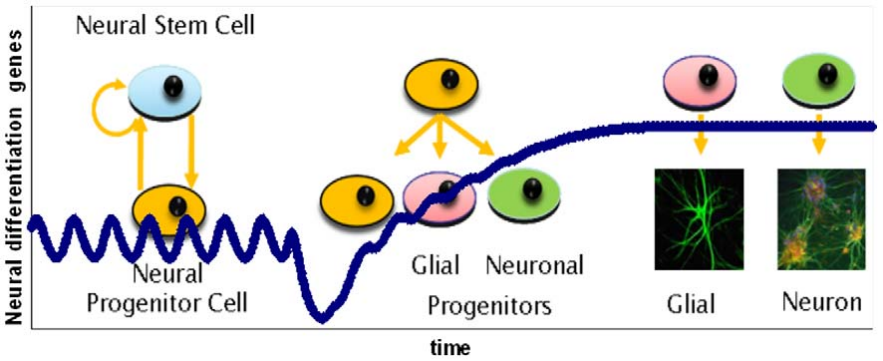
Dynamic evlolution of the pathway during commitment to a specific lineage and differentiation. The up-regulation of Mash1 is matched by an up-regulation in Hes6, which in turn further alleviates the inhibitory effects of Hes1. While the oscillations are dampened, asymmetric division is expected. The oscillatory behaviour induced by the Notch pathway will be alleviated after differentiation and the levels of expression should remain constant as the model describes.

The parameters (Table 3) identified by our model analysis as the most significant to the gene network were linearly altered by a factor of 10 one-at-a-time both in the absence and presence of a delta. Figure 8 depicts that not all of the parameters are capable of altering the behaviour of the network sufficiently so as to result in neural differentiation. Figure [9] qualitatively describes the expected dynamic transition phase of cells from the self renewal state towards differentiation. This has been associated with a dampening in the oscillations of Hes1 and Mash1 and a concomitant up regulation of Mash1 and down regulation of Hes1 [13]. [50] reports that the presence of Hes1 protein even in small amounts is enough to prohibit neural differentiation. Especially under the repressive effect of a delta signal received from an adjacent differentiating cell no parameter on its own was capable of altering the network towards neural differentiation. Consequently we examined the effect of these parameters on the behaviour of the network when altered simultaneously in pairs.

### The combined effect of varying two parameters simultaneously

**Figure 10:**
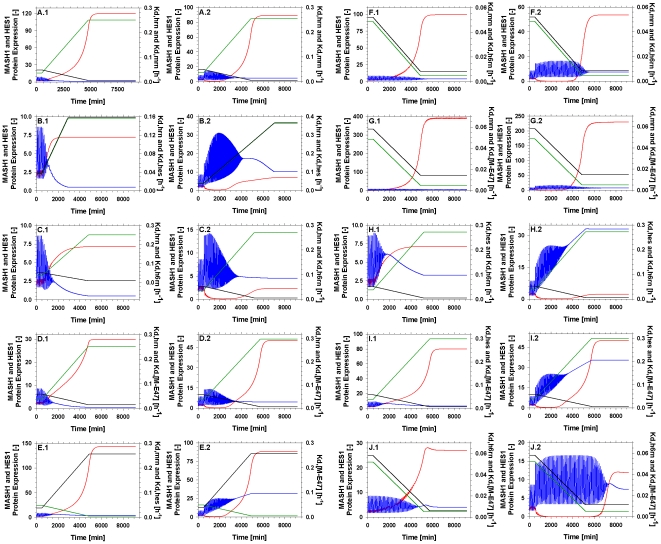
Dynamic evolution of the pathway while varying the value of the parameters of table 3 in pairs linearly by a factor of 10 both in the absence (subscripted with 1) and the presence (subscripted with 2) of a delta signal. The parameter values are plotted on the right axis and are denoted as (g) for the green line and (b) for the black line. The effect of an: (A) increase in the value of kd,HRN (g) and a decrease in the value of kd,MRN (b); (B) increase in the value of k_d,HRN_ (g) and an increase in the value of k_d,HES_ (b); (C) increase in the value of k_d,HRN_ (g) and a decrease in the value of k_d,H6RN_ (b); (D) increase in the value of k_d,HRN_ (g) and a decrease in the value of k_d,[M-E47]_ (b); (E) decrease in the value of k_d,MRN_ (g) and an increase in the value of k_d,HES_ (b); (F) decrease in the value of k_d,MRN_ (g) and a decrease in the value of k_d,H6RN_ (b); (G) decrease in the value of k_d,MRN_ (g) and a decrease in the value of k_d,[M-E47]_ (b); (H) increase in the value of k_d,HES_ (g) and a decrease in the value of k_d,H6RN_ (b); (I) increase in the value of k_d,HES_ (g) and a decrease in the value of k_d,[M-E47]_ (b); (J) decrease in the value of k_d,H6RN_ (g) and a decrease in the value of k_d,[M-E47]_ (b).

Figure 10 displays the results obtained when simultaneously varying two parameters at a time. In the absence of a repressive delta signal from a differentiating neighbouring cell almost all combinations of parameters are potent enough to induce the behaviour associated with neural differentiation. The only combination of model parameters that doesn't achieve an adequate repression of Hes1 in the absence of a delta signal is the pair *k_d,hes_ – k_d,h6rn_* (Figure 10.H.1). When varied one-at-a-time these two parameters (Figure 8.C.1 and 8.E.1) had the weakest overall effect on the gene network, therefore the shortcoming of this pairing is somewhat expected. However in the presence of a delta signal only 3 parameter pairs are able to reproduce behaviour resembling a differentiated state, summarised in Table 4. These pairs involve only 3 parameters and their possible combinations (*k_d,hrn_, k_d,mrn_, k_d,[M-E47]_*) further highlighting the crucial effect these parameters have on the behaviour of the modelled gene network. Our model based hypotheses generation approach presented herein, has initially highlighted the need for a conformational change within the Delta/Notch pathway in order to reach a resting state resembling differentiated cells and more importantly through model analysis has identified the parameters that could mediate such an effect.

**Table 4:** Parameters able to mediate neural differentiation.

Parameter	Appears in Figure:
k_d,HRN_	8.A, 10.A–D
k_d,MRN_	8.B, 10A, 10.E–G
k_d,[M-E47]_	8.E, 10.D,10.G,10.I–J

## Discussion

The Delta1/Notch1 signalling pathway has drawn scientific attention due to its significance during embryogenesis and development. A number of studies have modelled parts of the pathway and its main characteristic, which is an oscillation with a period of roughly 2 h. Herein, we have introduced a mathematical formulation that involves all the significant elements participating in the pathway, facilitating a more holistic description of the dynamic behaviour of the pathway. By studying the response of the pathway to disturbances introduced in some of its key components, namely Hes1, Mash1 and Hes6 protein concentrations we conclude that a functional change is required in order for the pathway's behaviour to shift towards neural differentiation, instigated perhaps through cross-talk with other pathways.

Even though this is not the first attempt to model the transition period of differentiating neural stem cells [18] we have attempted to link this transition with a plausible mechanism. Several studies, both model and experiment based, seem to concur to the fact that the altered behaviour in the Delta1/Notch1 pathway that leads to neural stem cell differentiation is instigated by an extrinsic, to the pathway, signal most probably as a result of cross-talk with other pathways [1], [2], [6], [20]. In reality more than one environmental parameter might lead cells to differentiation [3], [6], [7], [23]. In order to further look into this hypothesis we carried out a detailed model analysis and identified the partition of the parameter vector that accounts for the majority of the uncertainty in the model output. Subsequently we studied how a variation in the significant parameters would affect the behaviour of the pathway when varied one-at-a-time and in pairs of two. After a number of *in silico* experiments we identified three parameters (Table 4) as the most suitable candidates to mediate the behavioural change required for neural differentiation.

In agreement with current literature [4], [11], our model predicts a phase of gradually dampened oscillations, which corresponds to the period of asymmetric division before the pathway reaches its new resting state as a differentiated cell. Despite the qualitative nature of the model, valuable conclusions can be drawn. We posed and tested the hypothesis that even though the participants of the Notch signalling pathway determine cell fate, the participation of this pathway alone is not enough to induce differentiation. A detailed model analysis combined with *in silico* experimentation lead to the identification of the three most suitable candidates that can propagate the external, to the delta/Notch pathway, signal required for neural differentiation. It is now up to the experimentalists to verify the validity of our model driven hypothesis generation approach.

## Supporting Information

Supplemental Material S1 Derivative Based Global Sensitivity Measures and the derivation of the empirical cost function.
(0.10 MB DOC)

Figure S1 Phase planes of Hes1 (A–D), Mash1(E–H) and Hes6 (I–L) mRNA transcripts versus respective protein expression as a response to the application of a delta signal from a differentiating neighbouring cell for varying time periods. (A,E,I): Steady state (no delta signal); (B,F,J): 120 min application; (C,G,K): 240 min application; (D,H,L): 480 min application.
(3.11 MB TIF)

Figure S2 Phase planes of Hes1 (A–D), Mash1(E–H) and Hes6 (I–L) mRNA transcripts versus respective protein expression as a response to the application of variably sized pulses in the concentration of Hes1 for 960 min: (A,E,I): Steady state (no pulse); (B,F,J): 1× pulse; (C,G,K): 5× pulse; (D,H,L): 10× pulse.
(3.13 MB TIF)

Figure S3 Phase planes of Hes1 (A–D), Mash1(E–H) and Hes6 (I–L) mRNA transcripts versus respective protein expression as a response to the application of variably sized pulses in the concentration of Mash1 for 960 min: (A,E,I): Steady state (no pulse); (B,F,J): 1× pulse; (C,G,K): 5× pulse; (D,H,L): 10× pulse.
(3.16 MB TIF)

Figure S4 Phase planes of Hes1 (A–D), Mash1(E–H) and Hes6 (I–L) mRNA transcripts versus respective protein expression as a response to the application of variably sized pulses in the concentration of Hes6 for 960 min: (A,E,I): Steady state (no pulse); (B,F,J): 1× pulse; (C,G,K): 5× pulse; (D,H,L): 10× pulse.
(3.13 MB TIF)
