# Supplementary material for: Modelling the Delta1/Notch1 Pathway: In Search of the Mediator(s) of Neural Stem Cell Differentiation
Source: PLoS One. 2011 Feb 8;6(2):e14668. doi: 10.1371/journal.pone.0014668 (PMC3035613; doi:10.1371/journal.pone.0014668)
Supplement: Supplemental Material S1 — Derivative Based Global Sensitivity Measures and the derivation of the empirical cost function. (0.10 MB DOC) [file pone.0014668.s001.doc]

**Supplemental Material S1**

**Derivative Based Global Sensitivity Measures (DGSM)**

For a more detailed presentation of the method the reader is kindly referred to the original work of [41]. Let us consider a differentiable function$f\left(x\right)$, where $x=\{x_{i}\}$ is a vector of input variables defined within the unit hypercube . Local sensitivity measures are based on partial derivatives and are of the form:

$E_{i}(x*)=\frac{\partial f}{\partial x_{i}}$ (SM.1)

Sensitivity measure $E_{i}(x*)$ depends on a nominal point $x*$ and its value varies according to the value of$x*$. This deficiency can be overcome by averaging $E_{i}(x*)$ over the parameter space$I^{n}$. Such a measure can be defined as:

$\bar{M}_{i}=\int_{I^{n}} E_{i}dx$ (SM.2)

Another informative measure to consider is the variance of $\bar{M}_{i}$ which can be estimated by:

(SM.3)

$\bar{\Sigma}_{i}$ can also be presented as:

(SM.3b)

By combining the presented measures $\bar{M}_{i}$ and $\bar{\Sigma}_{i}$ a new measure can be derived.

(SM.4)

A normalised version of the new measure can be defined as:

$\bar{G}=\sum_{i=1}^{s} G_{i}/\sum_{i=1}^{n} G_{i}$, (SM.5)

Where 1 ≤ s ≤ n. The $\bar{G}$ measure can account for the fractional significance of a particular parameter with respect to the total variance, or for a group of parameters with respect to the total variance.

From (SM.4) we can obtain the ratio:

(SM.6)

Non-monotonic functions consist of regions with both positive and negative values of partial derivatives$E_{i}(x*)$, hence due to the effect of averaging, values of $\bar{M}_{i}$ can be very small or even zero. To avoid such situations measures based on the absolute value of $\left|E_{i}(x*)\right|$ can be used:

$\bar{M}_{i}^{*}=\int_{I^{n}} \left|E_{i}\right|dx$ (SM.7)

(SM.8)

Calculation of DGSM is based on the evaluation of integrals (SM.2)-(SM.8), which can be presented in the following generic form:

$I[f]=\int_{I^{n}} f(x)dx$ (SM.9)

It is assumed that function $f(x)$ is integrable in the *n*-dimensional unit hypercube I^n^.

The efficiency of MC methods is determined by the properties of random numbers. It is known that random number sampling is prone to clustering: for any sampling there are always empty areas as well as regions in which random points are wasted due to clustering. As new points are added randomly, they do not necessarily fill the gaps between already sampled points. A higher rate of convergence can be obtained by using deterministic uniformly distributed sequences also known as low-discrepancy sequences (LDS) instead of pseudo-random numbers, namely a QMC method. The QMC algorithm for the evaluation of the integral (SM.9) yields:

$I_{N}=\frac{1}{N}\sum_{i=1}^{N} f(q_{i}).$ (SM.10)

Here (q_i_) is a set of LDS points uniformly distributed in the unit hypercube. Evaluation of DGSM measures requires calculation of E_i_(x*). This can be done analytically for easy-differentiable functions or numerically:

$E_{i}\left(x^{*}\right)=\frac{\left[f\left(x_{1}^{*},\ldots,x_{i-1}^{*},x_{i}^{*}+\delta,x_{i+1}^{*},\ldots,x_{n}^{*}\right)-f\left(x^{*}\right)\right]}{\delta}$ (SM.11)

Here δ is a small increment. The total number of function evaluations required for the calculation of a full set of $\{\bar{M}_{i}\}$ and $\{\bar{\Sigma}_{i}\}$ is *N_F_* = *N* (*n* +1). Throughout this work we have used Sobol’ (LP-τ) quasi-random sequences in order to generate our sets of quasi-random points.

**Formulation of the empirical cost function**

The function used to evaluate the performance of the various randomly generated parameter vectors consists of various components each aimed to describe a qualitative experimental feature in a quantitative manner. The general form of the cost function can be found in equation (SM.12) below:

$\Delta=\sum_{j=1}^{6} w_{j}\delta_{j}$ (SM.12)

Where, w_j_ denote weighting factors for each component and δ_j_ denote the various summands each describing one qualitative feature. More specifically, a term penalising oscillations with a period different than 120min was added via equation (SM.13):

(SM.13)

Where *i* denotes either Hes1 or Mash1 concentration. In order to ensure that a steady oscillatory state had been reached within the studied time frame two terms penalising large standard deviations in period of oscillation and absence of oscillatory behaviour were added (SM.14 and SM.15)

$\delta_{2}=\sum_{i} \frac{std(\tau_{i})}{\varepsilon+average(\tau_{i})}$ (SM.14)

$\delta_{3}=\sum_{i} \frac{E}{\varepsilon+numberofpeaks_{i}}$ (SM.14)

Where ε is a sufficiently small number added to avoid issues arising with near zero values of the denominators and E is a scaling factor (herein E was equal to 10). Similarly two terms penalising a small or varied peak height were added (SM.16 and SM.17):

$\delta_{4}=\sum_{i} \frac{E}{\varepsilon+average(h_{i})}$ (SM.16)

$\delta_{5}=\sum_{i} \frac{std(h_{i})}{\varepsilon+average(h_{i})}$ (SM.17)

Finally a term penalising the cases were Mash1 concentration was larger than Hes1 concentration was added in the form of a binary function:

$\delta_{6}=\left\langle\begin{matrix} 0\begin{matrix} & \begin{matrix} for & \left(Mash1-Hes1\right)\leq0 \end{matrix} \end{matrix} \\ 100\begin{matrix} & \begin{matrix} for & \left(Mash1-Hes1\right)\geq0 \end{matrix} \end{matrix} \end{matrix}\right.$ (SM.18)

**Supplemental Figure Legends**

**Figure S1** Phase planes of Hes1 (A-D), Mash1(E-H) and Hes6 (I-L) mRNA transcripts versus respective protein expression as a response to the application of a delta signal from a differentiating neighbouring cell for varying time periods. (A,E,I): Steady state (no delta signal); (B,F,J): 120 min application; (C,G,K): 240 min application; (D,H,L): 480 min application.

**Figure S2** Phase planes of Hes1 (A-D), Mash1(E-H) and Hes6 (I-L) mRNA transcripts versus respective protein expression as a response to the application of variably sized pulses in the concentration of Hes1 for 960 min: (A,E,I): Steady state (no pulse); (B,F,J): 1x pulse; (C,G,K): 5x pulse; (D,H,L): 10x pulse.

**Figure S3** Phase planes of Hes1 (A-D), Mash1(E-H) and Hes6 (I-L) mRNA transcripts versus respective protein expression as a response to the application of variably sized pulses in the concentration of Mash1 for 960 min: (A,E,I): Steady state (no pulse); (B,F,J): 1x pulse; (C,G,K): 5x pulse; (D,H,L): 10x pulse.

**Figure S4** Phase planes of Hes1 (A-D), Mash1(E-H) and Hes6 (I-L) mRNA transcripts versus respective protein expression as a response to the application of variably sized pulses in the concentration of Hes6 for 960 min: (A,E,I): Steady state (no pulse); (B,F,J): 1x pulse; (C,G,K): 5x pulse; (D,H,L): 10x pulse.
